# A comparison of FDG PET/MR and PET/CT for staging, response assessment, and prognostic imaging biomarkers in lymphoma

**DOI:** 10.1007/s00277-022-04789-9

**Published:** 2022-02-16

**Authors:** Trine Husby, Håkon Johansen, Trond Bogsrud, Kari Vekseth Hustad, Birte Veslemøy Evensen, Ronald Boellard, Guro F. Giskeødegård, Unn-Merete Fagerli, Live Eikenes

**Affiliations:** 1 Department of Circulation and Medical Imaging, Faculty of Medicine and Health Sciences, Norwegian University of Science and Technology, Postboks 8905, Trondheim, Norway; 2 Department of Oncology, St. Olavs Hospital, Trondheim University Hospital, Trondheim, Norway; 3 Department of Radiology and Nuclear Medicine, St. Olavs Hospital, Trondheim University Hospital, Trondheim, Norway; 4 PET-Centre, University Hospital of North Norway, Tromsø, Norway; 5 Aarhus University Hosipital, Aarhus, Denmark; 6 Department of Nuclear Medicine and Molecular Imaging, University Medical Center Groningen, Groningen, The Netherlands; 7 Department of Radiology and Nuclear Medicine, Cancer Center Amsterdam, University Medical Centers Amsterdam, VUMC, Amsterdam, The Netherlands; 8 Department of Public Health and Nursing, Faculty of Medicine and Health Sciences, Norwegian University of Science and Technology, Trondheim, Norway; 9 Department of Clinical and Molecular Medicine, Faculty of Medicine and Health Sciences, Norwegian University of Science and Technology, Trondheim, Norway

**Keywords:** PET/MR, Lymphoma, Metabolic tumor volume, Deauville score

## Abstract

The aim of the current study was to investigate the diagnostic performance of FDG PET/MR compared to PET/CT in a patient cohort including Hodgkins lymphoma, diffuse large B-cell lymphoma, and high-grade B-cell lymphoma at baseline and response assessment. Sixty-one patients were examined with FDG PET/CT directly followed by PET/MR. Images were read by two pairs of nuclear medicine physicians and radiologists. Concordance for lymphoma involvement between PET/MR and the reference standard PET/CT was assessed at baseline and response assessment. Correlation of prognostic biomarkers Deauville score, criteria of response, SUVmax, SUVpeak, and MTV was performed between PET/MR and PET/CT. Baseline FDG PET/MR showed a sensitivity of 92.5% and a specificity 97.9% compared to the reference standard PET/CT (*κ* 0.91) for nodal sites. For extranodal sites, a sensitivity of 80.4% and a specificity of 99.5% were found (*κ* 0.84). Concordance in Ann Arbor was found in 57 of 61 patients (*κ* 0.92). Discrepancies were due to misclassification of region and not lesion detection. In response assessment, a sensitivity of 100% and a specificity 99.9% for all sites combined were found (*κ* 0.92). There was a perfect agreement on Deauville scores 4 and 5 and criteria of response between the two modalities. Intraclass correlation coefficient (ICC) for SUVmax, SUVpeak, and MTV values showed excellent reliability (ICC > 0.9). FDG PET/MR is a reliable alternative to PET/CT in this patient population, both in terms of lesion detection at baseline staging and response assessment, and for quantitative prognostic imaging biomarkers.

## Introduction

The goal of treatment for Hodgkins lymphoma (HL), diffuse large B-cell lymphoma (DLBCL), and high-grade B-cell lymphoma is most often cure. Accurate staging at baseline and response assessment is therefore essential for optimal treatment strategies. Furthermore, risk stratification and treatment decisions require reliable prognostic imaging biomarkers in addition to pretreatment clinical risk assessment scores.

It is well established that functional imaging with positron emission tomography (PET)-computed tomography (CT) with ^18^F-Fluorodeoxyglucose (FDG) is the standard imaging modality for FDG-avid lymphomas. Response adaptive FDG PET/CT guided treatment approach to evaluate chemosensitivity and to guide decision of radiotherapy allows for individual treatment strategies in HL [1, 2]. Furthermore, end of treatment response FDG PET/CT for detecting residual disease in DLBCL is an important prognostic factor and guides indication for consolidation radiotherapy [3].

PET/magnetic resonance (MR) has the advantage of simultaneous PET and magnetic resonance imaging (MRI) data acquisitions, combining metabolic activity from PET with great soft tissue contrast and functional data from MRI with diffusion weighted imaging (DWI). In addition, reduction of the radiation dose by eliminating the contribution from CT is of value for the young patient.

Although FDG PET/MR has shown comparable ability to PET/CT for lesion detection and anatomical staging in terms of Ann Arbor in a few studies in adult lymphoma populations [4–6], these studies have included highly heterogeneous lymphoma populations acquired at different time points of imaging evaluation. Only one recent study [7] has assessed a large, homogenous lymphoma population (HL) at baseline but lacks response assessment evaluations.

To establish whether FDG PET/MR is a reliable alternative to PET/CT in the care of lymphoma patients, there is also a need to compare the quantitative PET metrics between the two modalities. PET-detector technology and attenuation correction of the PET images differs between PET/CT and PET/MR, which can have a significant impact on the quantitative PET measurements.

Baseline maximum standardized uptake value (SUVmax) was one of the first quantitative PET measurements used as a prognostic biomarker, and studies have found that SUVmax from FDG PET/MR versus PET/CT correlates well [4–6, 8]. The role of baseline SUVmax in predicting treatment outcome is however discordant, and other quantitative PET metrics such as metabolic tumor volume (MTV) are reported in PET studies with increasing frequency. MTV is a promising and robust PET-based prognostic factor in HL [9, 10] and DLBCL [11], but no studies have yet compared MTV from FDG PET/MR and PET/CT in lymphoma patients.

Deauville score and PET-based criteria of response are other strong prognostic factors used in FDG PET/CT-based response assessment that guides treatment decisions. Deauville score (5-point scale) is based on visual assessment and SUV in residual lymphoma lesions compared with SUV in mediastinal blood pool and liver [12]. PET-based criteria of response [13] use Deauville score and change in SUV from baseline. The correlation of Deauville score between the FDG PET/CT and PET/MR has only been evaluated in one pediatric HL study [14], where they found excellent agreement, while no studies has compared criteria of response.

The aim for this prospective study was to investigate the diagnostic performance of FDG PET/MR with DWI compared to today’s standard PET/CT during first line treatment in a patient cohort including classical HL, DLBCL, and high-grade B-cell lymphoma. Primary endpoints were region-based and patient-based (Ann Arbor) agreement. Secondary endpoints were correlation of prognostic biomarkers in terms of Deauville score, criteria of response, SUVmax, SUVpeak, and MTV using FDG PET/CT as reference standard.

## Materials and methods

### Study population

Patients were enrolled from the lymphoma section at St. Olavs Hospital, Trondheim University Hospital, from June 2016 to February 2019. Sixty-four patients fulfilled the following inclusion criteria: 18 years or older, histological confirmed DLBCL, classical HL, or high-grade B cell lymphoma. Exclusion criteria were contra-indications for MRI or pregnancy. Three patients were excluded due to missing PET/MR raw data at baseline. This left 61 patients included in the study (Table 1).

**Table 1:** Study patient population

Patient population	*n* (%)
Age, median (range)	60 (22–82)
Gender *n* = 61	
Male	38 (62)
Female	23 (38)
Histology	
Classical HL	25 (41)
DLBCL	31 (51)
High-grade B-cell lymphoma	5 (8)
Performace status	
0–1	53 (87)
2–4	8 (13)
Ann Arbor stage	
I	3 (5)
II	23 (38)
III	8 (13)
IV	27 (44)
Bulky tumor	30 (49)
Prognostic score aggressive NHL *n* = 36	
IPI 0	2 (6)
IPI 1–2	16 (44)
IPI 3–5	18 (50)
Prognostic score cHL limited disease *n* = 13	
Favorable	2 (15)
Unfavorable	11 (85)
Prognostic score cHL advanced disease *n* = 12	
IPS 0–2	8 (67)
IPS ≥ 3	4 (33)

*IPI*, International Prognostic Index; *IPS*, International Prognostic Score in Hodgkins lymphoma; *HL*, Hodgkins lymphoma; *DLBCL*, diffuse large B-cell lymphoma.

All 61 patients were imaged with FDG PET/CT directly followed by PET/MR at baseline. Interim (after 2 cycles of chemotherapy only for HL) and end of treatment (3–6 weeks after chemotherapy for both HL and aggressive non-Hodgkin lymhoma (NHL)) examinations were performed on a subgroup of the patients when PET/CT was clinically indicated. A total of 108 (61 baseline, 13 interim and 34 end of treatment) FDG PET/MR and PET/CT examinations were therefore included in the study. The study was approved by the Regional Committee for Ethics in Medical Research (REK-Midt #2014/1289). All participants gave written informed consent before participation.

### Image acquisition

PET/CT and PET/MR data was acquired by using a single intravenous injection of ^18^F-FDG (4 MBq/kg). PET/CT and PET/MR were acquired 60 (median) minutes (range 59–64) and 100 (median) minutes (range 87–150) after injection, respectively. All patients fasted for at least 6 h before injection of ^18^F-FDG, and blood glucose levels were measured prior to radiotracer administration. None of the patients had hyperglycemia (> 10 mmol/L).

### PET/CT

PET/CT was acquired on a Siemens Biograph mCT (Simens Helthcare, Erlangen, Germany). Patients were examined with their arms up in 4–9 bed positions (depending on body height), 2.5–3 min per bed position (depending on body weight), covering top of skull to upper thighs. Non-contrast-enhanced, low-dose CT with 120 kV, 0.5 s rotation time, pitch 0.95, and 40 mAS was performed for attenuation correction and morphological correlation.

### PET/MR

A PET/MR system (Siemens Biograph mMR, Erlangen, Germany) was used for simultaneous PET and MRI acquisitions. Patients were examined with their arms down in 5 bed positions covering top of skull to upper thighs, 5 min for each bed position. Simultaneous MRI was acquired with the following MRI sequences: coronal T1 Dixon-Vibe, transversal diffusion-weighted MRI (DWI) (*b* values 50 and 800), transversal T2-HASTE, and coronal T2-TIRM. Breath-hold imaging was used for bed positions 2–4, covering the thorax and abdomen. Attenuation correction maps were calculated from the T1 Dixon-Vibe sequence, segmenting four tissue types (air, soft tissue, fat, and lung) into predefined linear attenuation coefficients.

### PET reconstructions

PET image reconstruction was performed with iterative reconstruction (3D OSEM algorithm, 3 iterations, 21 subsets, and 4-mm Gaussian filter) with point spread function (PSF) and decay-, attenuation-, and scatter-correction. Time-of-flight was used on PET/CT but was not available on the PET/MR system. A 400 × 400 matrix was used on the PET/CT while a 344 × 344 matrix was used on the PET/MR (this corresponds to a relatively similar pixel size on the two scanners).

### Image analyses

The PET/CT and PET/MR images were read by two pairs of nuclear medicine physicians (7 and 24 years of experience) and radiologists (13 and 14 years of experience) using the same standardized reading protocols. The nuclear medicine physicians interpreted the PET images and the radiologists interpreted the CT/MRI images separately, followed by a joint report for the PET/CT and PET/MR by each reading team. The readers were blinded for clinical status, but aware of the histology. To avoid recollection, a period of 4 weeks was required between readings from different modalities. Baseline images were available when reading interim and end of treatment scans. In case of disagreement between joint report on PET/CT or PET/MR between the two reading teams, a final consensus was made by a third group consisting of a clinician with access to biopsy results, primary staging, and follow-up results and one reader from each reading team. Standard clinical software, Syngo.Via (Simens Healthineers) and AW server (GE Healthcare) were used.

Lymph node regions were defined according to the Rey symposium [15]: cervical, axillary, infraclavicular, mediastinal, hilar, periaortic, messentary, pelvic, and inguinal femoral regions. Spleen, tonsils, and Waldeyers ring were separate lymphatic regions accessed by the readers. Extranodal regions were defined as follows: liver, lung, adrenal gland, breast, gastrointestinal tract, pancreas, salivary glands, thyroid, soft tissue/pleura, kidneys, skin, bladder, and brain.

Anatomical staging in terms of extent of lymphoma disease with the modified Ann Arbor staging system [13] was performed separately by a lymphoma oncologist based on the PET/CT and PET/MR readings in the study.

### PET reading

The Lugano Classification [13] criteria for staging and response assessment were used for the PET reading. Diffuse uptake in the spleen without focal lesions had to be higher than 150% of SUV liver to be classified as diffusely involved [10]. For response assessment, Deauville score of 5 was defined as ≥3 times greater than liver. SUVmax and SUVpeak were recorded in all disease regions at baseline, and in response assessment, SUVmax was measured in the tumor with highest uptake.

### Metabolic tumor volume

MTV was computed using the research software ACCURATE, a semi-automatic software tool for quantitative analysis of PET/CT [16]. Both nuclear medicine physicians segmented MTV independently on baseline PET/MR and PET/CT scans for the patients with aggressive NHL. Initially, an automated analysis was done with fixed SUV-threshold of 4.0 [17] before physiological uptake was excluded from the volume.

### CT and MR reading

On CT and MRI, a lymph node > 15 mm in largest diameter on axial sequences was defined as pathological for lymphoma involvement. Morphological criteria for splenic involvement were focal lesions or craniocaudal diameter more than 13 cm on coronal CT or MRI. Bulky tumor was defined as ≥ 10 cm in largest diameter. The reading of different MRI sequences was performed simultaneously with no distinct order to combine morphological and structural information.

### Statistical analyses

Inter-observer agreement between the two reading teams was assessed by kappa statistics for nominal categorical variables and weighted kappa score for ordinal categorical variables. Kappa values were indicative of poor (*k* < 0.2), fair (*k* 0.2–0.4), moderate (*k* > 0.4–0.6), good (*k* > 0.6–0.8), and excellent (*k* > 0.8) agreement [18]. The kappa values and the 95% confidence intervals were calculated for nodal and extranodal sites (lymphoma involvement or not) combined and disease stage (Ann Arbor I–II versus III–IV). Weighted kappa statistics were used for Deauville score (1–3, 4, or 5) and criteria of response (complete metabolic response, partial metabolic response, no metabolic response, or progressive metabolic response). Intraclass correlation coefficients (ICC) were used for inter-observer agreement for the continues variable MTV. ICC estimates less than 0.5, between 0.5 and 0.75, between 0.75 and 0.9, and greater than 0.9, which indicates poor, moderate, good, and excellent reliability, respectively [19].

Concordance for lymphoma involvement between consensus FDG PET/MR and the reference standard consensus PET/CT was assessed using kappa statistics, observed agreement, positive predictive values (PPV), negative predictive values (NPV), sensitivity and specificity at baseline, and response assessment scans (interim and end of treatment response). ICC estimates and their 95% confidence interval were calculated using absolute agreement and two-way mixed-effects model for continuous variables (SUVmax, SUVpeak, and MTV). ICC measurements showed no correlation (below 0.1) between measurements from multiple lesions in the same patient both on PET/CT and PET/MR; thus, no correction was performed when including measurements from multiple lesions in the same patients.

Additionally, Bland–Altman plots [20] were made to evaluate the bias between the mean differences of SUVmax and MTV measured on FDG PET/MR and PET/CT. The difference of SUVmax between PET/MR and PET/CT was not normally distributed, and a nonparametric approach was therefore used by employing the median and inter-quartile range to estimate the limits of agreement (LoA) (± 1.45 *IQR) in the Bland–Altman plot. The difference of MTV was normally distributed, and a parametric method was used in terms of the mean difference and the standard deviation to estimate the LoA (mean ± 1.96 SD of the difference) in the Bland–Altman plot. All statistical analyses were performed using SPSS version 26.0.

## Results

### Inter-observer agreement

Inter-observer agreement between the two reading teams was overall good to excellent for both PET/CT and PET/MR (Table 2). The kappa agreement for nodal sites combined was *κ* 0.94 for PET/CT (95% CI 0.88–1) and *κ* 0.93 for PET/MR (95% CI 0.85–0.99). Kappa agreement of extranodal sites combined was *κ* 0.91 for PET/CT (95% CI 0.82–0.99) and *κ* 0.96 for PET/MR (95% CI 0.89–1). Weighted kappa agreement on Deauville score 1–3, 4, or 5 was good for PET/CT *κ* 0.76 (95% CI 0.51–1) and excellent *κ* 0.86 (95% CI 0.66–1) for PET/MR. Inter-observer agreement on disease stage in terms of Ann Arbor I–II versus III–IV was on PET/CT *κ* 0.89 (95% CI 0.79–1) and *κ* 0.93 (95% CI 0.84–1) on PET/MR. For criteria of response, the inter-observer weighted kappa agreement was good, PET/CT *κ* 0.73 (95%CI 0.41–1) and *κ* 0.75 (95%CI 0.47–1) on PET/MR. ICC showed excellent reliability for MTV measurement between the two readers on both PET/CT 0.96 (0.93–0.98) and PET/MR 0.96 (0.92–0.98) (*p* < 0.001).

**Table 2:** Inter-observer agreement between the two reading teams on FDG PET/CT and PET/MR

Inter-observer agreement	Kappa value (95% CI)	
	PET/CT	PET/MR
Nodal sites combined	0.94 (0.88–1)	0.93 (0.85–0.99)
Extranodal sites combined	0.91 (0.82–0.99)	0.96 (0.89–1)
Deauville score 1–3/4–5*	0.76 (0.51–1)	0.86 (0.66–1)
Ann Arbor I–II/III–IV	0.89 (0.79–1)	0.93 (0.84–1)
Criteria of response*	0.73 (0.41–1)	0.75 (0.47–1)
	ICC (95% CI)	
MTV	0.96 (0.93–0.98)	0.96 (0.92–0.98)

*Weighted kappa.

### Consensus FDG PET/MR vs. PET/CT at baseline

**Fig. 1:**
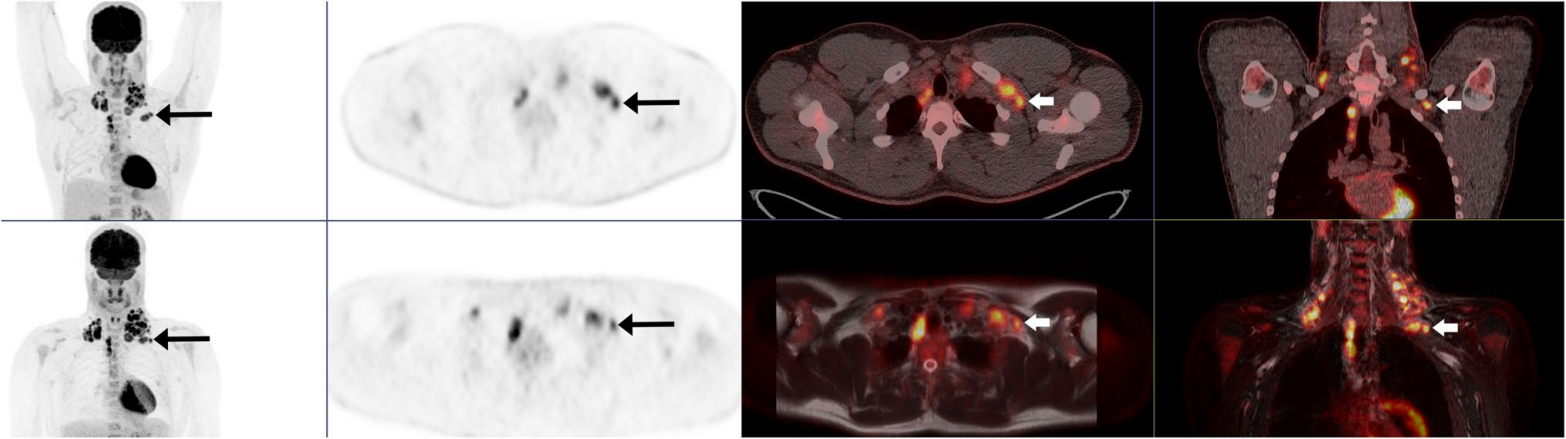
A 29-year-old male with classical HL stage IIB. Baseline PET/CT (arms up) in the upper row and PET/MR (arms down) in the bottom row. In consensus PET/MR, the left infraclavicular FDG avid lymph node was classified as left cervical, but scored as left infraclavicular on PET/CT

**Fig. 2:**
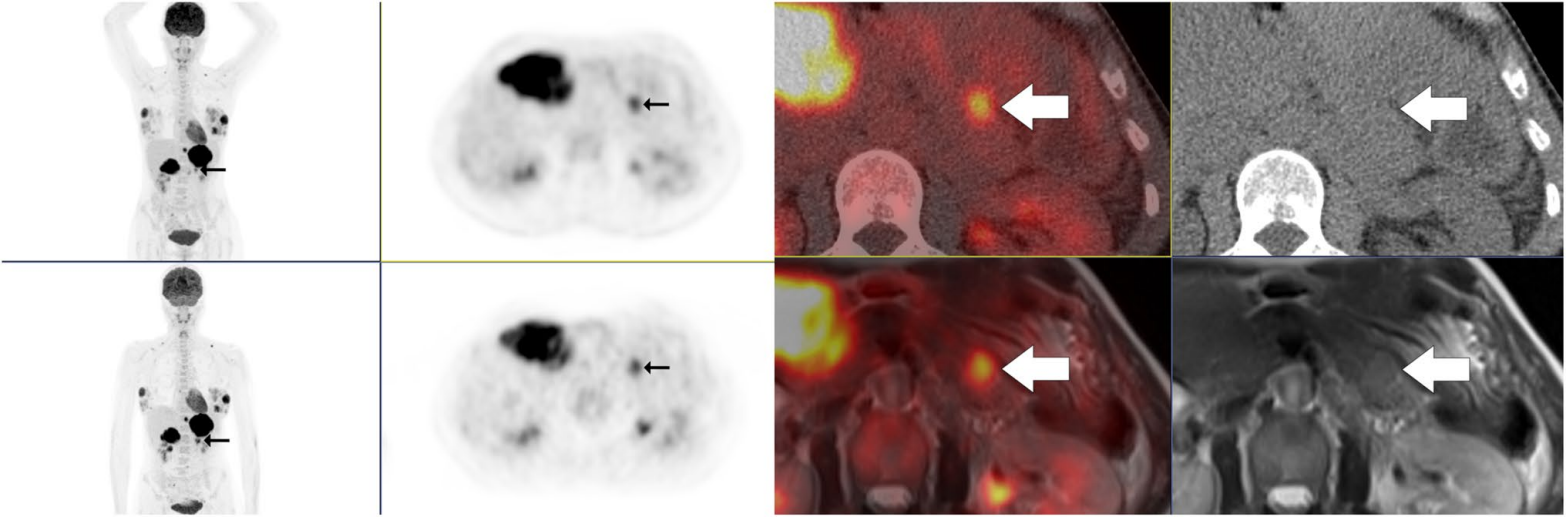
A 48-year-old female with DLBCL stage IVA. Baseline PET/CT in the upper row and PET/MR in the bottom row. On PET/MR T2-HASTE, we see a 18 × 14 mm solid lesion in cauda pancreatic that was scored as a paraaortic lymph node on PET/CT

**Fig. 3:**
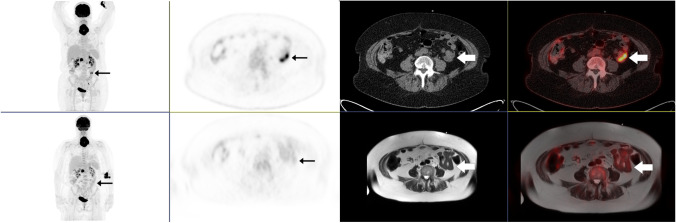
A 60-year-old female with DLBCL stage IVAX. Baseline PET/CT in the upper row and PET/MR in the bottom row. PET/CT shows a distinct FDG uptake in the small intestine, histological verified with biopsy from ileum. The lesion is not detectable on PET/MR

When comparing consensus baseline FDG PET/MR with PET/CT as the reference standard (Table 3) for nodal sites combined, an observed agreement was found in 1001/1037 sites (96.5%), with a sensitivity of 92.5%, specificity 97.9%, PPV 93.9%, and NPV 97.4%, and the kappa value showed excellent agreement *κ* 0.91 (95% CI 0.88–0.94). Of the 36 discrepant nodal sites, 16 were false positive on PET/MR with PET/CT as the reference standard [cervical (3), infraclavicular (4), pelvic (2), hilar (4), mesenteric (1), spleen (1), and Waldeyers ring (1)] and 20 were false negatives [axillary (1), femoral (1), infraclavicular (5), mediastinal (4), hilar (4), mesenteric (2), periaortic (1), spleen (1), and tonsils (1)]. An example of such a false-positive nodal site on PET/MR is shown in Fig. 1, where a left infraclavicular FDG avid lymph node was classified the as left cervical on PET/MR, but scored as left infraclavicular on PET/CT. For extranodal sites combined, observed agreement was found in 960/976 sites (98.3%), with sensitivity 80.4%, specificity 99.5%, PPV 90.0%, and NPV 98.8%, and the kappa value showed excellent agreement *κ* 0.84 (95% CI 0.76–0.92). Of the 16 discrepant extranodal sites, 5 were false positive on PET/MR compared with the PET/CT [bone marrow (1), pancreas (2), salivary gland (1), and kidney (1)]. An example of a false-positive extranodal site on PET/MR is shown in Fig. 2, where PET/MR demonstrates a compelling radiological FDG avid lesion in pancreas that was scored as a paraaortic lymph node on PET/CT. Eleven extranodal sites were false negative on PET/MR compared with PET/CT [bone marrow (2), adrenal glands (2), GI tract (1), pancreas (1), thyroid (1), and soft tissue including pleura (4)]. Figure 3 shows an example of a false-negative lesion in the small intestine on PET/MR compared to a distinct FDG uptake on PET/CT, which was also histologically confirmed with biopsy from ileum.

**Table 3:** Consensus FDG PET/MR versus consensus PET/CT at baseline staging for our study population in terms of nodal and extranodal sites separate and combined and Ann Arbor staging

Site	Observed agreement	Sensitivity	Specificity	PPV	NPV	Kappa value (95% CI)
Right cervical	59/61	100.0	91.3	95.0	100.0	0.93 (0.83–1)
Left cervical	60/61	100.0	96.4	97.1	100.0	0.97 (0.9–1)
Right axillary	60/61	90.0	100.0	100.0	98.1	0.94 (0.82–1)
Left axillary	61/61	100.0	100.0	100.0	100.0	1 (NA)
Right femoral	61/61	100.0	100.0	100.0	100.0	1 (NA)
Left femoral	60/61	85.7	100.0	100.0	98.2	0.91 (0.75–1)
Right infraclavicular	56/61	66.7	96.2	75.0	94.3	0.66 (0.38–0.93)
Left infraclavicular	57/61	75.0	96.2	75.0	96.2	0.71 (0.45–0.98)
Right pelvic	60/61	100.0	97.7	94.4	100.0	0.96 (0.88–1)
Left pelvic	60/61	100.0	97.9	92.9	100.0	0.95 (0.86–1)
Mediastinal	57/61	88.2	100.0	100.0	87.1	0.87 (0.75–0.99)
Hilar	53/61	78.9	90.5	78.9	90.5	0.69 (0.5–0.89)
Mesenteric	58/61	88.9	97.7	94.1	95.5	0.88 (0.75–1)
Periaortic	60/61	95.7	100.0	100.0	97.4	0.97 (0.9–1)
Spleen	59/61	92.3	97.9	92.3	97.9	0.9 (0.77–1)
Waldeyers ring	60/61	100.0	98.3	66.7	100.0	0.79 (0.4–1)
Tonsils	60/61	83.3	100.0	100.0	98.2	0.9 (0.71–1)
Bone marrow	58/61	85.7	97.9	92.3	95.8	0.86 (0.7–1)
Liver	61/61	100.0	100.0	100.0	100.0	1 (NA)
Lung	61/61	100.0	100.0	100.0	100.0	1 (NA)
Adrenal gland	59/61	33.3	100.0	100.0	96.7	0.49 (− 0.11–1)
Breast	61/61	100.0	100.0	100.0	100.0	1 (NA)
Gastrointestinal tract	60/61	80.0	100.0	100.0	98.2	0.88 (0.65–1)
Pancreas	58/61	50.0	96.6	33.3	98.3	0.38 (− 0.18–0.93)
Salivary glands	60/61	100.0	98.3	66.7	100.0	0.79 (0.4–1)
Thyroid	60/61	50.0	100.0	100.0	98.3	0.66 (0.04–1)
Soft tissue/pleura	57/61	55.6	100.0	100.0	92.9	0.68 (0.39–0.97)
Kidneys	60/61	100.0	98.3	50.0	100.0	0.66 (0.04–1)
Ann Arbor I–II/III–IV	59/61	NA	NA	NA	NA	0.93 (0.84–1)
Ann Arbor I–IV**	57/61	NA	NA	NA	NA	0.92 (0.85–1)
Nodal sites combined	1001/1037	92.5	97.9	93.9	97.4	0.91 (0.88–0.94)
Extranodal sites combined	960/976	80.4	99.5	90.0	98.8	0.84 (0.76–0.92)
All sites combined	1961/2013	90.4	98.8	93.3	98.2	0.9 (0.88–0.93)

*Skin, genitalia, brain, and bladder: no disease

**Weighted kappa

*NA*, not applicable

We found concordance in disease stage (Ann Arbor 0–IV) in 57/61 patients (93%) with a weighted kappa value of *κ* 0.92 (95% CI 0.85–1). When comparing limited disease, I–II versus extended disease III–IV, 59/61 was staged similar on PET/MR and PET/CT.

### Consensus FDG PET/MR vs. PET/CT for response assessment

Table 4 shows consensus interim and end of treatment FDG PET/MR compared with PET/CT as the reference standard. Observed agreement for nodal sites combined was 798/799 (99.9%), with a sensitivity 100%, specificity 99.9%, PPV 87.5%, and NPV100%. The discrepant was a false-positive axillary node on PET/MR. For extranodal sites, the observed agreement was 751/752 (99.8%), sensitivity 100%, specificity 99.9%, PPV 80.0%, and NPV 100%. One PET/MR examination graded the bone marrow as false positive compared to PET/CT.

**Table 4:** Consensus PET/MR versus consensus PET/CT response assessment (13 interim and 34 end of treatment scans) in terms of nodal and extranodal sites separate and combined. Deauville score and criteria of response

Site	Observed agreement	Sensitivity	Specificity	PPV	NPV	Kappa value (95% CI)
Right cervical	47/47	100.0	100.0	100.0	100.0	1 (NA)
Left cervical	47/47	100.0	100.0	100.0	100.0	1 (NA)
Right axillary	47/47	100.0	100.0	100.0	100.0	1 (NA)
Left axillary	46/47	NA	97.9	NA	100.0	NA
Right femoral	47/47	NA	100.0	NA	100.0	NA
Left femoral	47/47	NA	100.0	NA	100.0	NA
Right infraclavicular	47/47	NA	100.0	NA	100.0	NA
Left infraclavicular	47/47	100.0	100.0	100.0	100.0	1 (NA)
Right pelvic	47/47	NA	100.0	NA	100.0	NA
Left pelvic	47/47	NA	100.0	NA	100.0	NA
Mediastinal	47/47	100.0	100.0	100.0	100.0	1 (NA)
Hilar	47/47	NA	100.0	NA	100.0	NA
Mesenteric	47/47	NA	100.0	NA	100.0	NA
Periaortic	47/47	100.0	100.0	100.0	100.0	1 (NA)
Spleen	47/47	NA	100.0	NA	100.0	NA
Waldeyers ring	47/47	NA	100.0	NA	100.0	NA
Tonsils	47/47	NA	100.0	NA	100.0	NA
Bone marrow	46/47	100.0	97.8	50.0	100.0	0.66 (0.03–1)
Liver	47/47	100.0	100.0	100.0	100.0	1 (NA)
Lung	47/47	NA	100.0	NA	100.0	NA
Adrenal gland	47/47	NA	100.0	NA	100.0	NA
Breast	47/47	NA	100.0	NA	100.0	NA
Gastrointestinal tract	47/47	NA	100.0	NA	100.0	NA
Pancreas	47/47	NA	100.0	NA	100.0	NA
Salivary glands	47/47	NA	100.0	NA	100.0	NA
Thyroid	47/47	NA	100.0	NA	100.0	NA
Soft tissue/pleura	47/47	100.0	100.0	100.0	100.0	1 (NA)
Kidneys	47/47	NA	100.0	NA	100.0	NA
Deauville score 1–3/4–5	47/47	NA	NA	NA	NA	1 (NA)
Deauville score 1–5**	41/47	NA	NA	NA	NA	0.72 (0.54–0.89)
Criteria of response**	47/47	NA	NA	NA	NA	1 (NA)
Nodal sites combined	798/799	100.0	99.9	87.5	100.0	0.93 (0.8–1)
Extranodal sites combined	751/752	100.0	99.9	80.0	100.0	0.89 (0.67–1)
All sites combined	1549/1551	100.0	99.9	84.6	100.0	0.92 (0.8–1)

*Skin, genitalia, brain, and bladder: no disease

**Weighted kappa

*NA*, not applicable due to no true positive lymphoma lesions on PET/CT and no false negative on PET/MR or one of the variables are constant.

**Fig. 4:**
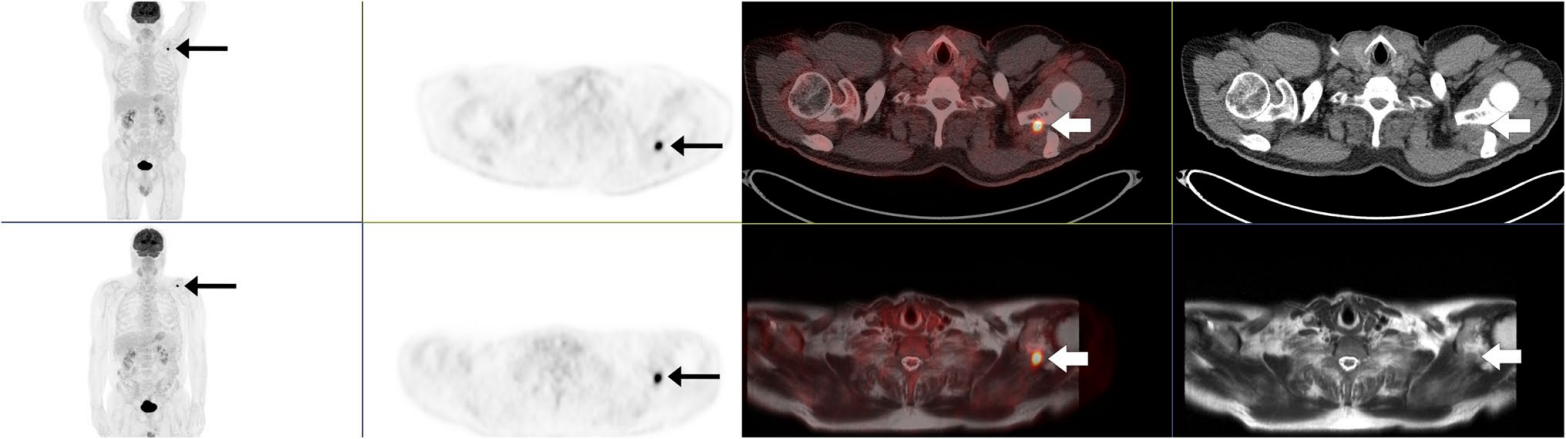
A 71-year-old male with high-grade B-cell lymphoma stage IVA. End of treatment PET/CT in the upper row and PET/MR in the bottom row. The FDG avid lesion was scored as soft tissue on PET/CT and bone marrow on PET/MR. The lesion was localized in scapulae in the MRI sequences T2-HASTE, DWI (b800), ADC map, and T1Dixon in phase. Deauville score 5 and partial metabolic response on both modalities

All of the 47 consensus interim and end of treatment examinations were scored similar on FDG PET/MR and PET/CT in terms of criteria of respons. Deauville score grading showed good agreement on weighted kappa, *κ* 0.72 (95% CI 0.54–0.89). Difference in Deauville score grading between PET/MR and PET/CT was seen in 11 patients and only in those with complete metabolic response (Deauville score 1–3). Figure 4 shows a patient with partial metabolic response and Deauville score 5 at end of treatment response on both image modalities, while the FDG avid lesion was scored as soft tissue on PET/CT but correctly identified in scapula on PET/MR.

### SUV values at baseline

**Fig. 5:**
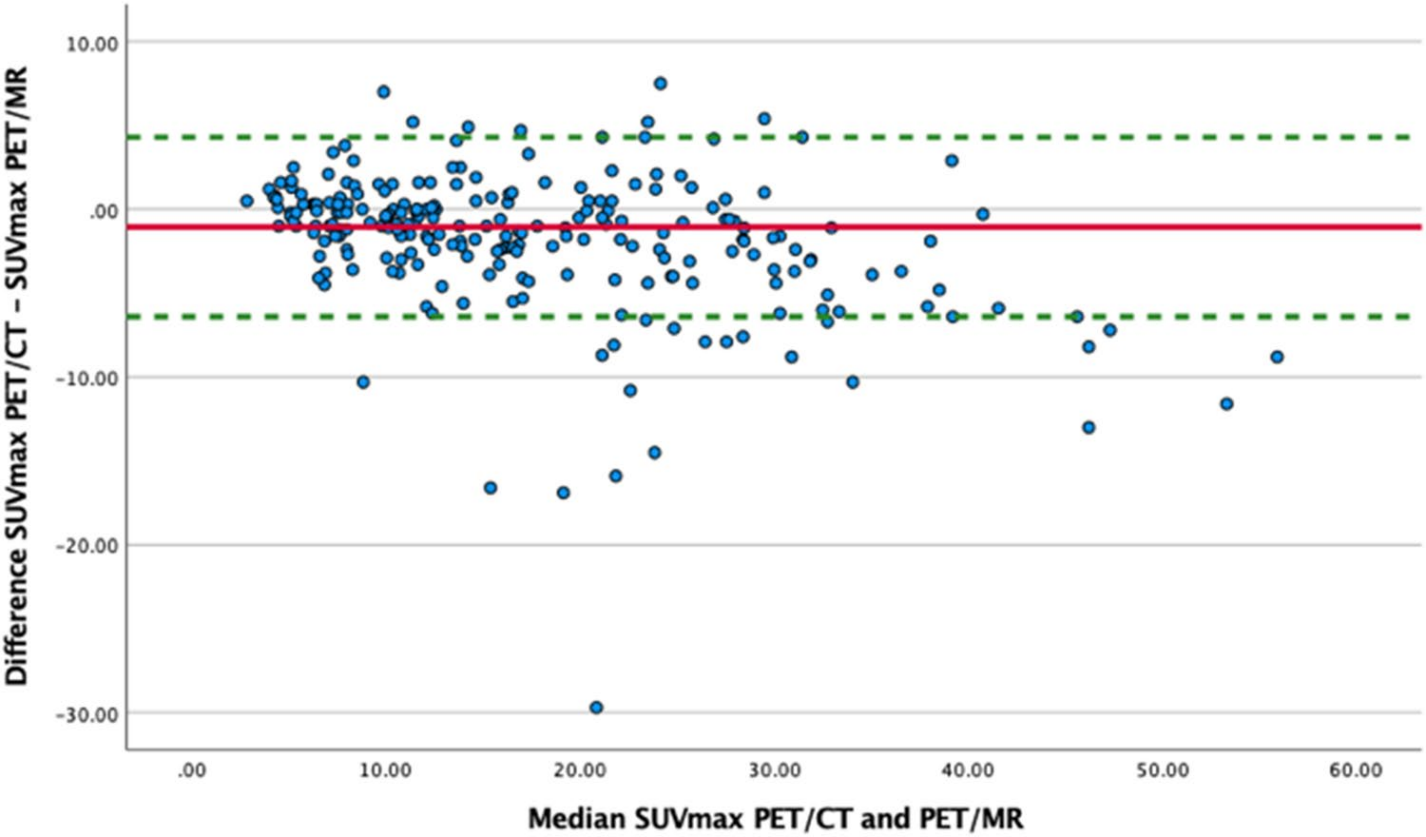
Bland-Altmann plot of maximum standardized uptake value (SUVmax) in 236 lymphoma lesions measured on both PET/MR and PET/CT. The red, solid line shows median difference in SUV max − 1.05 (IQR = 3.90) (higher median SUVmax on PET/MR than with PET/CT) with 95% limits of agreement, green dotted line − 6.4 to 4.3

ICC was calculated for SUVmax (*n* = 236) and SUVpeak values (*n* = 235) from lymphoma lesions detected on both modalities. ICC estimates and their 95% confident intervals were 0.95 (0.91–0.97) for SUVmax and 0.96 (0.94–0.97) for SUVpeak, both showing excellent reliability (*p* < 0.001). The Bland-Altmann plot of SUVmax (Fig. 5) showed median difference of − 1.05 (LoA − 6.4 to 4.3) demonstrating slightly higher median SUVmax on PET/MR than PET/CT.

### Metabolic tumor volume at baseline

**Fig. 6:**
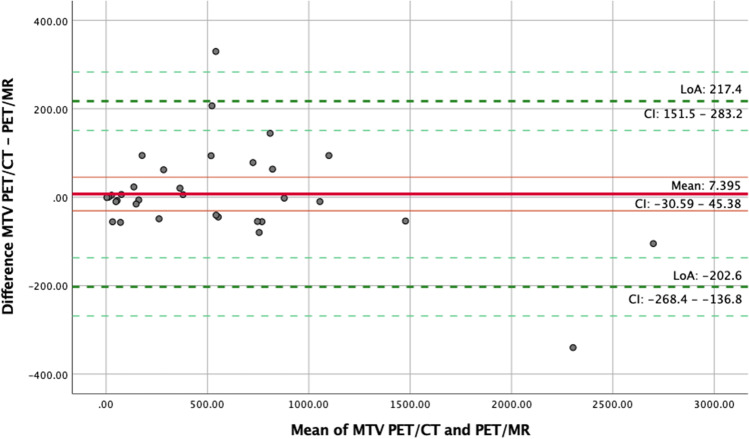
Bland-Altmann plot of metabolic tumor volume (MTV) in 33 patients with DLBCL or high-grade B-cell lymphoma at baseline PET/MR and PET/CT. The red, solid lines show mean difference in MTV (7.4, higher on PET/CT than PET/MR) and its confidence interval. Green dotted lines present 95% limits of agreement, − 206.2 to 217.4 and their confidence interval

MTV was measured in 33 patients with DLBCL or high-grade B-cell lymphoma at baseline. Two patients were not included in this analysis due to no detectable disease on both modalities, and one was excluded because it was impossible to delineate lymphoma tissue form kidney and bladder on both PET modalities. ICC estimate and the 95% confidence intervals were 0.99 (0.98–1) for MTV, showing excellent reliability (*p* < 0.001). A Bland-Altmann plot of MTV (Fig. 6) showed a slightly higher MTV with PET/CT than with PET/MR, with a mean difference of 7.4 (LoA − 202.6 to 217.4).

## Discussion

In this prospective study, we have compared FDG PET/MR to today’s standard PET/CT at baseline and response assessment in 61 patients with classical HL, DLBCL, or high-grade B-cell lymphoma.

Excellent kappa agreement was found for lymphoma staging of nodal sites combined when comparing consensus FDG PET/MR versus PET/CT with a sensitivity of 92.5% and a specificity of 97.9%. The nodal regions with most discrepancies were infraclavicular, hilar, and mediastinal lymph nodes. Previous studies comparing MRI versus FDG PET/CT for lymphoma staging have also found these nodal regions most challenging [21, 22]. In all cases of discrepancies in the current study, both modalities showed the same FDG avid lesions, but the readers scored the lymph node region different between FDG PET/MR and PET/CT. Most likely, the explanation on the labeling difference in the infraclavicular region is the positioning of the arms, which differs between PET/CT (arms up) and PET/MR (arms down) (Fig. 1). Motion artifacts and poor quality of the DWI sequences were reported in one of the misclassifications of hilar and mediastinal nodal regions. A few of the discrepancies can be explained by higher SUV value on FDG PET/CT than on PET/MR and vice versa and therefore classified as reactive lymph node on one of the modalities.

For staging of extranodal sites combined, excellent kappa agreement between consensus FDG PET/MR and PET/CT was also found, but with a lower overall sensitivity and specificity for PET/MR. The exstranodal regions with most discrepancies were pancreas, bone marrow, and soft tissue/pleura. All, except one (Fig. 3), of the lesions were visible on both modalities, and the main reason for the discrepancies was different interpretation of location of the lesions, as illustrated in Fig. 2. Other lymphoma studies have found a higher concordance of extranodal disease between FDG PET/MR and PET/CT [6, 8, 14]. However, compared to these studies, our patient population had a higher burden of extranodal disease, and in contrast to other study designs [22], we did not correct reader errors before the statistical analyses. This approach was chosen to reflect clinical imaging reading.

Concordance in disease stage (Ann Arbor) was found in 57 of 61 patients. Three patients were understaged and one upstaged with FDG PET/MR versus PET/CT. The reason for under staging on FDG PET/MR was two patients with soft tissue or pleural involvement on PET/CT classified as nodal lesions on PET/MR, and one periaortic node with lower SUVmax on PET/MR and therefore interpreted as a reactive node. The discrepancy that led to upstaging on FDG PET/MR was a cervical lymph node with higher SUVmax on FDG PET/MR, interpreted as reactive node on PET/CT. In our cohort, this would have had clinical treatment consequences in one HL patient if staged with FDG PET/MR instead of PET/CT.

In response assessment scans, we found a sensitivity of 100% and a specificity of 99.9% for FDG PET/MR on both nodal and extranodal sites combined. Seven of the 43 response assessment scans had FDG avid disease present on both modalities. Among these patients, one patient with partial metabolic response on both examinations also had an axillary node that was graded positive on FDG PET/MR due to higher SUVmax than on PET/CT. In addition, one bone marrow was graded positive on FDG PET/MR and labeled as soft tissue on PET/CT (Fig. 4).

A recent study of pediatric HL patients found excellent agreement on Deauville score grading between FDG PET/MR and PET/CT [14]. Our study also showed a perfect agreement on Deauville score 4 and 5 between the two modalities. Differences were only found in patients with complete metabolic response (Deauville score 1–3) and would not have altered any treatment decisions. The reason for this difference is hard to postulate due to no observable trend in the results. In addition, all consensus response assessment scans were graded similar on FDG PET/MR and PET/CT in terms of criteria of respons, meaning that none of the included patients would have been treated any different based on FDG PET/MR response assessment examinations versus PET/CT.

When studying SUVmax and SUVpeak, both showed excellent reliability, but a slightly higher median SUVmax was found on FDG PET/MR compared to PET/CT. These findings may relate to the prolonged uptake time on PET/MR in our study (median 100 min after injection), as increased SUV is reported in lymphoma patients until 2 h after FDG injection [23]. Previous lymphoma studies acquiring PET/CT before PET/MR has however demonstrated both higher SUVmax [5] and lower SUVmax [8, 14] on PET/MR, indicating that the differences in SUV between the modalities are caused by other factors such as heterogeneity in the included patients in the studies or technical differences between the sites.

To our knowledge, this is the first study to compare MTV between FDG PET/CT and PET/MR in lymphoma patients. Our results showed excellent reliability in baseline MTV in 33 patients with aggressive NHL. MTV was slightly higher with FDG PET/CT than with PET/MR with a mean difference of 7.4 cm^3^. When considering the scale of MTV values in our population, both the mean difference and the LoA indicate good agreement among most of the patients and no systematic bias of clinical importance. There was no misclassification in attenuation correction maps or other external explanations in the images with the largest differences. MTV has previously been compared between FDG PET/CT and PET/MR retrospectively in a lung and pancreatic cancer population [24] and the authors found that a threshold of SUV2.5 was more robust against imaging modality and protocol compared to SUV50%. Our study did however only use one method for calculating MTV, which could be a limitation. There is controversy regarding which method to use for calculating MTV, and ongoing work is done in order to standardize MTV measurements worldwide [25]. However, although the use of various contouring thresholds, such as SUV2.5, SUV4.0, and 41% of SUVmax, results in significantly different MTV values for the same PET-data, the various MTV methods have shown to predict prognosis with similar accuracy [25, 26]. For that reason, only one method was used to calculate MTV in the current study. A recent study found that SUV4.0 was one of the two best methods for calculating MTV in lymphoma patients [17]. Together with our results, this study supports the use of SUV4.0 as a robust method for calculating MTV.

A notable limitation of our study is that all the FDG PET/MR scans was performed after PET/CT. Future studies may scan half of the patients with PET/MR first to even out the difference in time of administration of FDG to time of imaging. Furthermore, the lack of a diagnostic contrast-enhanced CT for the radiologist to use for anatomical correlation when interpreting FDG PET/CT may have affected the interpretation of extranodal lesion localizations on the reference standard.

In conclusion, our study shows an overall excellent agreement between FDG PET/MR and PET/CT in terms of lesion detection at baseline staging and response assessment for adult patients with classical HL, DLBCL, and high-grade B-cell lymphoma. The discrepancies between the modalities were mainly due to misclassification of region and not due to lesion detection. The PET-based quantitative prognostic imaging biomarkers also showed good agreement between the two modalities, demonstrating that FDG PET/MR is a reliable alternative to PET/CT in this patient population.

## Data Availability

The datasets generated and/or analyzed during the current study are not publicly available due to the European Union General Data Protection Regulations (GDPR), but are available from the corresponding author on reasonable request.
